# Prevalence of trypanosomiasis caused by *Trypanosoma evansi* (Kinetoplastea, *Trypanosomatidae*) in domestic ruminants from Southern Punjab, Pakistan

**DOI:** 10.14202/vetworld.2024.1955-1965

**Published:** 2024-09-01

**Authors:** Muhammad Tariq, Farhad Badshah, Muhammad Salman Khan, Eliana Ibáñez-Arancibia, Patricio R. De los Ríos-Escalante, Naimat Ullah Khan, Sadaf Naeem, Azka Manzoor, Rabia Tahir, Muhammad Mubashir, Muhammad Ilyas, Ghulam Ali Manzoor, Mourad Ben Said

**Affiliations:** 1 Department of Zoology, Cholistan University of Veterinary and Animal Sciences, Bahawalpur, Pakistan; 2 Department of Zoology, Abdul Wali Khan University, Mardan, Pakistan; 3 Ph.D. Program in Sciences mentioning Applied Molecular and Cell Biology, La Frontera University, Temuco, Chile; 4 Laboratory of Engineering, Biotechnology and Applied Biochemistry, Department of Chemical Engineering, Faculty of Engineering and Science, La Frontera University, Temuco, Chile; 5 Department of Biological and Chemical Sciences, Faculty of Natural Resources, Catholic University of Temuco, Temuco, Chile; 6 Nucleus of Environmental Sciences, Faculty of Natural Resources, Catholic University of Temuco, Temuco, Chile; 7 Collage of Veterinary Science, Abdul Wali Khan University Mardan, Mardan, Pakistan; 8 School of Life Science and Technology, Xi’an Jiaotong University, Xi’an 710049, China; 9 Collage of Veterinary and Animal Sciences, Islamia University of Bahawalpur, Bahawalpur, Pakistan; 10 College of Animal Science and Technology, Sichuan Agricultural University, Chengdu, 611130, China; 11 Department of Animal Nutrition, Cholistan University of Veterinary and Animal Sciences, Bahawalpur, Pakistan; 12 Directorate of Agriculture Research Transfer Technology, Mastung, Balochistan; 13 Department of Basic Sciences, Higher Institute of Biotechnology of Sidi Thabet, University of Manouba, Manouba 2010, Tunisia; 14 Laboratory of Microbiology, National School of Veterinary Medicine of Sidi Thabet, University of Manouba, Manouba 2010, Tunisia

**Keywords:** domestic ruminants, microscopic examination, Pakistan, polymerase chain reaction detection, *Trypanosoma evansi*, trypanosomiasis

## Abstract

**Background and Aim:**

Trypanosomiasis, a parasitic infection caused by various *Trypanosoma* species, poses a significant threat to global livestock, affecting both human health and economic sectors. This study aimed to estimate the prevalence of *Trypanosoma evansi* in Southern Punjab, Pakistan, focusing on key ruminant species, including camels, cattle, buffaloes, goats, and sheep.

**Materials and Methods:**

A total of 240 blood samples, comprising 48 samples from each animal species (camel, cattle, buffaloes, goat, and sheep) were collected from three districts in Southern Punjab. The collected samples were subjected to thin smear microscopy, DNA extraction, and polymerase chain reaction (PCR) amplification. The molecular characterization was conducted using the TBR primer set, which targeted repeated satellite DNA regions and the cytochrome oxidase II gene of *T*. *evansi*.

**Results:**

About 22.08% (53/240) of overall samples were positive for trypanosomiasis, with prevalence rates being 23.75% (19/80), 21.25% (17/80), and 21.75% (17/80) for districts Muzaffargarh, Lodhran, and Bahawalpur, respectively. 5.83% (14/240) of samples tested for *T. evansi* using PCR were positive in the districts of Muzaffargarh 7.50% (6/80), Lodhran 5.00% (4/80), and Bahawalpur 5.00% (4/80). Among the animals tested, camels had the highest positivity rate. The microscopic examination confirmed infection rates of 45.83% (22/48) for camels, 18.75% (9/48) for cattle, 8.33% (4/48) for buffaloes, 18.75% (9/48) for goats, and 18.75% (9/48) for sheep (p < 0.001). PCR results did not reveal substantial differences (p < 0.05) in prevalence: camels 12.50% (6/48), cattle 6.25% (3/48), buffaloes 0% (0/48), goats 8.33% (4/48), sheep 2.08% (1/48); while distinct disparities were detected district-wise: Muzaffargarh 23.75% (19/80), Lodhran 21.25% (17/80), and Bahawalpur 21.25% (17/80). The PCR results for these districts were insignificantly different: 7.50% (6/80), 5% (4/80), and 5% (4/80). The microscopic infection rate in camels from Bahawalpur was 56.30% (9/16). The microscopic analysis in Buffaloes reported a 6.30% (1/16) infection rate, but PCR results indicated no infections (0%) in any district. A significant difference (p < 0.001) in identifying *Trypanosoma* species was found between positively and negatively tested animals in both microscopic and PCR methods.

**Conclusion:**

This study emphasizes the necessity of regularly using PCR-based screening for its superior sensitivity and specificity over traditional microscopy. The varying occurrence of trypanosomiasis among districts reflects the intricate nature of this diseases epidemiology in the region. Reducing economic losses from trypanosomiasis in Southern Punjab, Pakistan, requires targeted interventions, such as vector control measures and farmer education.

## Introduction

The livestock industry significantly contributes value addition and national Gross domestic product (GDP) in Pakistan’s agricultural sector. In 2021, agriculture accounted for 60.1% of the value added and 11.5% of the overall GDP. Eight million rural residents earn around 35%–40% of their income from livestock. The budget allocation for the fiscal year 2020-21 witnessed an increase, reaching 8.55 billion USD compared to 8.30 billion USD in the preceding fiscal year. The livestock sector’s GDP contribution registered a 3.06% increase, according to Khan [1]. The livestock sector’s growth rate was limited to 3.1% in fiscal year 2021 due to an 8.9% increase in intermediate consumption. Approximately 1.5 million people are now employed in the livestock sector. Pakistan’s arid environment supports a substantial population of cows, which contributes significantly to the country’s livestock genetic diversity. According to the Government of Pakistan’s data from 2021, the country boasts 42.4 million buffaloes, 51.5 million cattle, 31.6 million sheep, 80.3 million goats, and 1.1 million camels [2].

In countries where cattle production is substantial, parasitism imposes significant economic losses. High parasite burdens in ruminants can result in increased mortality, weight loss, reduced fertility, and lower productivity [3–5]. Trypanosomiasis, a parasitic ailment, poses a pervasive threat to various animals worldwide, including cattle, buffaloes, sheep, goats, camels, donkeys, horses, mules, pigs, dogs, and cats [6]. These biting flies, such as tabanus, chrysops, atylotus, lyperosia, haematopota, and stomoxys transmit the disease mechanically. The disease presents with symptoms including intermittent fever, reduced appetite, excessive eye tearing, petechial hemorrhages in the conjunctiva, anemia, edema of the limbs and genitalia, swollen lymph nodes, miscarriage, impaired fertility, and weight loss, ultimately resulting in premature death in severe cases. Neurological symptoms, emaciation, and even fatality may ensue [7]. In endemic areas, trypanosomiasis brings about lower calving rates, less animal work, and higher calf fatalities [8].

The life cycle of trypanosomiasis is divided into two stages, occurring within both the tsetse fly and mammalian hosts. The infection begins when the tsetse fly introduces metacyclic parasites into the host’s skin through its saliva, leading to the formation of a chancre [9]. Subsequently, some parasites transform into elongated bloodstream trypomastigotes, undergo division, and generate shorter, stumpy bloodstream parasites [10]. The tsetse fly consumes the final stages during a blood meal. These forms have vital adaptations for surviving in the insect vector’s habitat. In the insect’s midgut, they transform into procyclic forms before migrating to and developing into epimastigotes in the salivary glands. Eventually, infectious metacyclic forms emerge, preadapted to thrive and reproduce in the bloodstream of mammals [11, 12]. The economic impact of trypanosomiasis is substantial, resulting in both direct and indirect losses. Direct losses include reductions in meat, milk, and manure production, animal mortality, abortion in pregnant females, and expenses associated with disease management programs and treatments [13]. Indirect losses arise from reduced production potential caused by trypanosomiasis. The treatment of trypanosomiasis is financially demanding, highlighting the need for a crucial cost-benefit analysis to assess the socioeconomic losses inflicted by the disease [14]. This analysis encompasses the costs associated with diagnosing and treating affected animals (veterinary services, medications, and operational expenses), chemoprophylaxis treatments, vector management programs, and research expenditures [15].

This study aimed to estimate the prevalence of *Trypanosoma evansi* in ruminants in southern Punjab, Pakistan. In different localities, prevalence rates in camels, cattle, buffaloes, goats, and sheep were identified using both microscopic and polymerase chain reaction (PCR) techniques, providing insight into its effect on livestock health.

## Materials and Methods

### Ethical approval

The study was approved by the ethical council of the Cholistan University of Veterinary and Animal Science (CUVAS), Cholistan, Pakistan. Blood samples from animals were collected by a trained person as per the standard sample collection without harming or giving unnecessary stress.

### Study period and location

**Figure-1:**
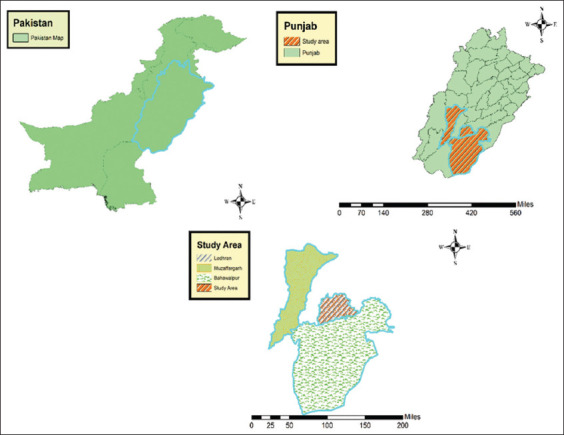
Map shows the three districts from where the study animals were sampled in Khyber Pakhtunkhwa, Pakistan [Source: The map was generated using ArcGIS 10.8.2].

The study was conducted from March 2021 to July 2022 in three districts in Southern Punjab, Pakistan, namely, Muzaffargarh, Lodhran, and Bahawalpur (Figure-1). The geographical coordinates for the districts are as follows: Muzaffargarh (30°4'27.7572" N, 71°11'4.7544" E), Lodhran (29°31'59.99" N, 71°37'59.99" E), and Bahawalpur (29°25'5.0448" N, 71°40'14.4660" E). These areas are characterized by a dry tropical climate.

### Sampling

A total of 240 blood samples were collected, with 48 samples from each ruminant species that were apparently healthy without any clinical symptoms, including *Camelus dromedarius* (camels)*, Bos taurus* (cattle)*, Bubalus bubalis* (buffaloes), *Capra aegagrus hircus* (goats), and *Ovis aries* (sheep). Blood samples were randomly acquired, irrespective of the sex and age of the animals. Blood samples (5 mL) were collected aseptically from the jugular vein using EDTA-containing vacutainer tubes (Thermo Fisher Scientific, USA) and transported on ice to the laboratory.

### Detection of Trypanosoma spp.

**Figure-2:**
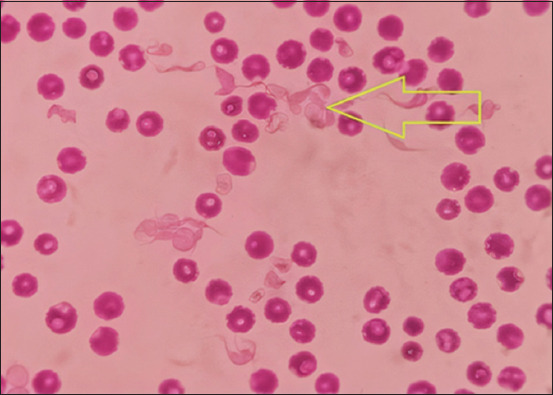
Morphological characteristics of *Trypanosoma* spp. revealed in a Giemsa-stained blood smear under a microscope.

Blood smears were prepared for *Trypanosoma* species’ detection. A drop of fresh blood was applied to a clean glass slide, spread with a second glass slide at a 45° angle, and fixed with absolute ethanol. Giemsa staining was performed, and the slides were examined using an oil immersion microscope (Nikon, USA) at a magnification of 100× (Figure-2). Following parasitological assessments, samples were stored at −20°C until PCR analysis.

### DNA extraction and PCR amplification

**Figure-3:**
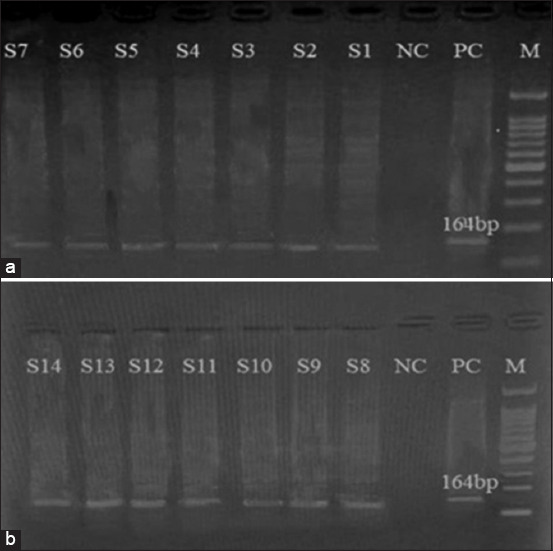
Polymerase chain reaction amplification results for specific detection of *Trypanosoma evansi* DNA based on TBR primers. Legend: a and b: Lane 1; 100 bp DNA ladder; Lane S1 to S14; Samples positive for *T. evansi*. Lane PC; positive control; Lane NC, negative control.

DNA extraction from the randomly selected samples was conducted using the Wiz prep^®^ Genomic DNA purification kit (Promega, USA). The amplification of maxicircle kinetoplast DNA (kDNA) from *T. evansi* was achieved using a pair of oligonucleotide primers, TBR 1 (5´-GAATATTAAACAATGCGCAG-3´) and TBR2 (5´-CCATTTATTAGCTTTGTTGC-3´), following the method described previously [16]. The PCR reaction was performed in a total volume of 25 μL consisting of the following components: 1 μL of DNA template at a concentration of 100 ng/μL, 1.25 μL each of forward and reverse primers at 10 μM concentration, 5 μL of 5X Q5 reaction buffer to achieve a final concentration of 1X, and 0.5 μL of a 10 mM dNTP mixture to provide a final concentration of 200 μM. The reaction included 0.25 μL of Q5 High-Fidelity DNA Polymerase (Fermentas, UK) to ensure high fidelity in DNA synthesis. The remaining volume was adjusted with 15.75 μL of DNase-free deionized water. Positive controls were established using blood samples from ruminants exhibiting clinical signs of trypanosomiasis, while water served as the negative control. A DNA thermal cycler (Gene Amp® PCR system 2700 Applied Biosystems Inc., UK) was employed for the amplification process. The thermal profile, adapted from Ijaz *et al*. [17], included an initial denaturation at 94°C for 10 min, followed by 35 cycles of denaturation at 94°C for 1 min, annealing at 55°C for 1 min, and elongation at 72°C for 1 min, with a final extension at 72°C for 10 min. PCR results were preserved at 4°C until electrophoresis separation on a 2.5% agarose gel and subsequent visualization under a UV Trans illuminator (Bio-Rad, USA) (Figure-3).

### Statistical analysis

The data obtained from this study were analyzed using descriptive statistics to estimate the prevalence of *Trypanosoma* infection across the study area. To assess the strength of associations between categorical variables, statistical tests such as the chi-square test and binomial test were employed. These tests evaluated the significance of associations between variables and determined if the observed associations were statistically significant. A confidence interval of 95% was used for the analyses [18]. The statistical analysis was performed using SPSS statistical software version 20.0 for Windows (IBM Corp., NY, USA). The significance level was set at p < 0.05. Results with p < 0.01 were considered highly significant.

## Results

### Ruminant-specific prevalence

Microscopic examination revealed an overall prevalence rate of 22.08% (53/240) for trypanosomiasis. Specifically, camels exhibited the highest prevalence rate, followed by goats, cattle, sheep, and buffaloes. Microscopic results indicated significant infection rates in camels 45.83% (22/48), cattle 18.75% (9/48), buffaloes 8.30% (4/48), goats 18.75% (9/48), and sheep (18.75%) (9/48) (p < 0.001). The overall PCR prevalence rate of trypanosomiasis was 5.80% (14/240). All the samples were determined through PCR amplification of maxicircle kDNA of *T. evansi* and exhibited varying rates across ruminant species. Specifically, the prevalence rate was 12.50% (6/48), 6.25% (3/48), 0.00% (0/48), 8.33% (4/48), and 2.08% (1/47) in camels, cattle, buffaloes, goats, and sheep, respectively. A chi-square test was employed for comparison between positive and negative categories concerning prevalence methods across different districts. Microscopic analysis revealed an infection rate of 22.10% (53/240) in all animals, with statistically significant differences (p < 0.001). Conversely, PCR yielded a total infection rate of 5.80% (14/240), demonstrating non-significant differences (p = 0.071). Breaking down the results by locality, microscopic analysis indicated infection rates of 23.75% (19/80) in Muzaffargarh, 21.25% (17/80) in Lodhran, and 21.25% (17/80) in Bahawalpur, with a total infection rate of 22.10% (53/240), all statistically non-significant (p = 0.908). PCR results showed infection rates of 7.50% (6/80) in Muzaffargarh, 5.00% (4/80) in Lodhran, and 5.00% (4/80) in Bahawalpur, contributing to a total infection rate of 5.83% (14/240), also statistically non-significant (p = 0.738).

### Microscopic and PCR analyses by ruminant type

Microscopic examination of 48 blood samples from each ruminant species across the three districts revealed prevalence rates of 45.83% (22/48) in camels, 18.75% (9/48) in cattle, 8.33% (4/48) in buffaloes, 18.75% (9/48) in goats, and 18.75% (9/48) in sheep. The overall microscopic infection rate across all animals was 22.10% (53/240), demonstrating statistical significance (p = 0.000). In contrast, PCR revealed infection rates of 12.50% (6/48) in camels, 6.25% (3/48) in cattle, 0.00% (0/48) in buffaloes, 8.33% (4/48) in goats, and 2.08% (1/48) in sheep. The total PCR infection rate was 5.80% (14/240), indicating non-significant differences (p = 0.071).

### Microscopic and PCR findings according to the districts

Microscopic analysis of samples from Muzaffargarh, Lodhran, and Bahawalpur exhibited infection rates of 23.75% (19/80), 21.25% (17/80), and 21.25% (17/80), respectively, with a total infection rate of 22.08% (53/240), showing no statistical significance (p = 0.908). Conversely, PCR results displayed prevalence rates of 7.50% (6/80), 5.00% (4/80), and 5.00% (4/80) for the respective districts, contributing to a total PCR infection rate of 5.83% (14/240), also with no statistical significance (p = 0.738) (Table-1).

**Table-1:** Risk factors analysis of *Trypanosoma* spp. infection by microscopic examination and *Trypanosoma evansi* infection by polymerase chain reaction assay in domestic ruminants from Pakistan.

Risk factors	Classes	Microscopic examination			Molecular identification		
	
		Positive/Total	Rate (% ± C.I.^1^)	p-value	Positive/Total	Rate (% ± C.I.^1^)	p-value
Ruminant types	Large	35/144	24.31 (0.173–0.313)	0.309	9/144	6.25 (0.028–0.109)	0.736
	Small	18/96	18.75 (0.113–0.261)		5/96	5.21 (0.017–0.119)	
Ruminant species	Cattle	9/48	18.75 (0.076–0.298)	0.000*	3/48	6.25 (0.001–0.170)	0.071
	Camel	22/48	45.83 (0.317–0.599)		6/48	12.50 (0.046–0.252)	
	Buffalo	4/48	8.33 (0.004–0.161)		0/48	0	
	Sheep	9/48	18.75 (0.076–0.298)		1/48	2.08 (0.001–0.109)	
	Goat	9/48	18.75 (0.076–0.298)		4/48	8.33 (0.022–0.204)	
District	Muzaffargarh	19/80	23.75 (0.151–0.347)	0.908	6/80	7.50 (0.028–0.158)	0.738
	Lodhran	17/80	21.25 (0.129–0.322)		4/80	5.00 (0.013–0.128)	
	Bahawalpur	17/80	21.25 (0.129–0.322)		4/80	5.00 (0.013–0.128)	
Total		53/240	22.10 (0.166–0.275)		14/240	5.83 (0.0286–0.0880)	

1 C.I.=95% confidence interval,

* Statistically significant, *P <* 0.05

### Microscopic and PCR outcomes in the large and small ruminants

Microscopic analysis indicated prevalence rates of 24.31% (35/144) in large ruminants and 18.75% (18/96) in small ruminants, with a total infection rate of 22.08% (53/240), demonstrating non-significant differences (p = 0.309). PCR results showed a prevalence rate of 6.25% (9/144) in large ruminants, 5.21% (5/96) in small ruminants, and a total PCR infection rate of 5.83% (14/240), again with non-significant differences (p = 0.736) across different localities (Table-1).

### District-wise microscopic and PCR findings

In district Muzaffargarah, microscopic prevalence rates were 37.50% (6/16) in camels, 25.0% (4/16) in cattle, 6.25% (1/16) in buffaloes, 18.75% (3/16) in goats, and 31.25% (5/16) in sheep. Total microscopic infection rate: 23.75% (19/80) and non-significant (p = 0.276). PCR infection rates: 6.25% (1/16) in camels, 12.50% (2/16) in cattle, 0.00% (0/16) in buffaloes, 12.50% (2/16) in goats, and 6.30% (1/16) in sheep. Total PCR infection rate: 7.50% (6/80) and non-significant (p = 0.641). In the Lodhran district, microscopic prevalence rates were 43.75% (7/16) in camels, 18.80% (3/16) in cattle, 12.50% (2/16) in buffaloes, 25.00% (4/16) in goats, and 6.30% (1/16) in sheep. Total microscopic infection rate: 21.30% (17/80) and non-significant (p = 0.095). PCR infection rates: 12.50% (2/16) in camels, 6.30% (1/16) in cattle, 0.00% (0/16) in buffaloes, 6.30% (1/16) in goats, and 0.00% (0/16) in sheep. Total PCR infection rate: 5.00% (4/80) and non-significant (p = 0.450). In Bahawalpur district, microscopic prevalence rates were 56.30% (9/16) in camels, 12.50% (2/16) in cattle, 6.30% (1/16) in buffaloes, 12.50% (2/16) in goats, and 18.80% (3/16) in sheep. Total microscopic infection rate: 21.30% (17/80), highly significant (p = 0.004). PCR infection rates: 18.80% (3/16) in camels, 0.00% (0/16) in cattle, 0.00% (0/16) in buffaloes, 6.30% (1/16) in goats, and 0.00% (0/16) in sheep. Total PCR infection rate: 5.00% (4/80) and non-significant (p = 0.062) (Table-1).

### Ruminant-specific prevalence across districts

For camels, microscopic examination revealed varied prevalence rates in Muzaffargarh (37.50%), Lodhran (43.75%), and Bahawalpur (56.25%), with an overall prevalence rate of 45.83% (22/48), indicating no statistical significance (p = 0.556). PCR displayed rates of 6.25% (1/16), 12.50% (2/16), and 18.75% (3/16) in Muzaffargarh, Lodhran, and Bahawalpur, contributing to a total prevalence rate of 12.50% (6/48), similarly showing no statistical significance (p = 0.565). For cattle, when considering the microscopic method, prevalence rates were 25% (4/16), 18.75% (3/16), and 12.50% (2/16) in Muzaffargarh, Lodhran, and Bahawalpur, respectively, resulting in an overall prevalence rate of 18.75% (9/48), with no statistical significance (p = 0.663). In contrast, PCR indicated rates of 12.50% (2/16), 6.25% (1/16), and 0.00% (0/16) in Muzaffargarh, Lodhran, and Bahawalpur, contributing to a total prevalence rate of 6.25% (3/48), with no statistical significance (p = 0.344). For buffaloes, prevalence rates were 6.25% (1/16), 12.50% (2/16), and 6.25% (1/16) in Muzaffargarh, Lodhran, and Bahawalpur, respectively, resulting in a total prevalence rate of 8.33% (4/48), demonstrating no statistical significance (p = 0.761). Conversely, PCR showed zero infection rates (0/16) in Muzaffargarh, Lodhran, and Bahawalpur, leading to a total prevalence rate of 0.00% (0/48), with high statistical significance (p = 0.000).

For goats, the microscopic method indicated infection rates of 18.75% (3/16), 25.0% (4/16), and 12.50% (2/16) in Muzaffargarh, Lodhran, and Bahawalpur, totaling 18.75% (9/48), showing no statistical significance (p = 0.663). PCR displayed rates of 12.50% (2/16), 6.25% (1/16), and 6.250% (1/16) in Muzaffargarh, Lodhran, and Bahawalpur, contributing to a total prevalence rate of 8.33% (4/48), similarly demonstrating no statistical significance (p = 0.761). For sheep, the microscopic method showcased infection rates of 31.25% (5/16), 6.25% (1/16), and 18.75% (3/16) in Muzaffargarh, Lodhran, and Bahawalpur, respectively, resulting in an overall prevalence rate of 18.75% (9/48), indicating no statistical significance (p = 0.194). On the other hand, PCR revealed rates of 6.25% (1/16), 0.00% (0/16), and 0.00% (0/16) in Muzaffargarh, Lodhran, and Bahawalpur, contributing to a total prevalence rate of 2.10% (1/48), similarly showing no statistical significance (p = 0.360) (Table-2).

**Table-2:** Chi-square test for the relationship between prevalence and districts with respect to methods (microscopic and polymerase chain reaction results) for different animals.

Animal	Methods	Muzaffargarh		Lodhran		Bahawalpur			
		
		Positive/Total	Rate (% ± C.I.^1^)	Positive/Total	Rate (% ± C.I.^1^) p-value	Positive/Total	Rate (% ± C.I.^1^)	Overall prevalence (%)	p-value
Camel	Microscopic	6/16	37.50 (0.1377–0.6122)	7/16	43.75 (0.187–0.712)	9/16	56.25 (0.292–0.804	22/48 (45.83)	0.556
	PCR	1/16	6.25 (0.002–0.329)	2/16	12.50 (0.016–0.386)	3/16	18.75 (0.039–0.459	6/48 (12/50)	0.556
Cattle	Microscopic	4/16	25.00 (0.073–0.524)	3/16	18.75 (0.039–0.459)	2/16	12.50 (0.016–0.386	9/48 (18.75)	0.663
	PCR	2/16	12.50 (0.016–0.386)	1/16	6.25 (0.002–0.329)	0/16	0.00 (0.000–0.216	3/48 (6.25)	0.344
Buffalo	Microscopic	1/16	6.25 (0.002–0.329)	2/16	12.50 (0.016–0.386)	1/16	6.25 (0.002–0.329	4/48 (8.33)	0.761
	PCR	0/16	0.00 (0.000–0.216)	0/16	0.00 (0.000–0.216)	0/16	0.00 (0.000–0.216	0//48 (0)	0.000
Goat	Microscopic	3/16	18.75 (0.039–0.459)	4/16	25.00 (0.073–0.524)	2/16	12.50 (0.016–0.386	9/48 (18.75)	0.663
	PCR	2/16	12.50 (0.016–0.386)	1/16	6.25 (0.002–0.329)	1/16	6.25 (0.002–0.329	4/48 (8.33)	0.761
Sheep	Microscopic	5/16	31.25 (0.107–0.588)	1/16	6.25 (0.002–0.329)	3/16	18.75 (0.039–0.459	9/48 (18.75)	0.194
	PCR	1/16	6.25 (0.002–0.329)	0/16	0.00 (0.000–0.216)	0/16	0.00 (0.000–0.216	1/48 (2.08)	0.360

C.I.=95% confidence interval, PCR=Polymerase chain reaction

### Microscopic and PCR findings across different ruminant species

For large ruminants**,** the microscopic results revealed an infection rate of 45.83% (22/48) in camels, 18.75% (9/48) in cattle, and 8.33% (4/48) in buffaloes. The overall infection rate was 24.31% (35/144) for all large ruminants, and these rates were statistically non-significant (p = 0.374) across all districts. For large ruminants, PCR results showed an infection rate of 12.50% (6/48) in camels, 6.25% (3/48) in cattle, and 0% (0/48) in buffaloes. The overall infection rate was 6.25% (9/144), and these rates were statistically non-significant (P=0.173) across all districts.

For small ruminants**,** microscopic results revealed an infection rate of 18.75% (9/48) in goats and 18.75% (9/48) in sheep. The overall infection rate for all small ruminants was 18.75% (18/96), which was statistically non-significant (p = 0.273) across all districts. In contrast, PCR results showed an infection rate of 8.33% (4/48) in goats and 2.08% (2/48) in sheep. The overall infection rate for all small ruminants was 6.25% (6/96), and these rates were statistically non-significant (p = 0.028) across all districts (Table-1).

### Binomial test analysis for all animals

The binomial test was used to compare positive and negative results for the microscopic and PCR methods across all animals, with details presented in Tables-3 and 4. Using the microscopic method, highly significant differences (p < 0.001) were observed in camels (22/48), cattle (9/48), buffaloes (4/48), goats (9/48), and sheep (9/48). Using the PCR assay, similar highly significant differences (p < 0.001) were noted in camels (6/48), cattle (3/48), buffaloes (0/48), goats (4/48), and sheep (1/48). The binomial test was also performed for each district, highlighting significant differences between positive and negative results. In Muzaffargarh, highly significant differences (p = 0.001) were observed in buffaloes (1/16) through microscopic analysis and in camels (1/16), cattle (2/16), buffaloes (0/16), goats (2/16), and sheep (1/16) through PCR. In Lodhran, highly significant differences (p = 0.000) were observed in buffaloes (2/16) and sheep (1/16) through microscopic analysis, whereas cattle (3/16) showed significant differences (p = 0.021). PCR analysis revealed that camels (2/16), cattle (1/16), buffaloes (0/16), goats (1/16), and sheep (0/16) displayed highly significant differences (p < 0.001). In Bahawalpur, highly significant differences (p < 0.001) were observed in cattle (2/16) and buffaloes (1/16), whereas sheep (3/16) showed significant differences through microscopic analysis. Through PCR analysis, cattle (0/16), buffaloes (0/16), goats (1/16), and sheep (0/16) displayed highly significant differences (p < 0.001), with camels (3/16) showing significant differences (Tables-3 and 4).

**Table-3:** Binomial test (proportion test) for comparison between positive and negative categories with respect to large ruminants and methods for different districts regarding prevalence.

Animal	Methods	Category	Muzaffargarh			Lodhran			Bahawalpur		
		
			Prevalence	Proportion	p-value	Prevalence	Proportion	p-value	Prevalence	Proportion	p-value
Camel	Microscopic	Positive	6	0.38	0.454^NS^	7	0.44	0.804^NS^	9	0.56	0.804^NS^
		Negative	10	0.63		9	0.56		7	0.44	
		Total	16	1.00		16	1.00		16	1.00	
	PCR	Positive	1	0.06	0.001**	2	0.13	0.004**	3	0.19	0.021*
		Negative	15	0.94		14	0.88		13	0.81	
		Total	16	1.00		16	1.00		16	1.00	
Cattle	Microscopic	Positive	4	0.25	0.077^NS^	3	0.19	0.021*	2	0.13	0.004**
		Negative	12	0.75		13	0.81		14	0.88	
		Total	16	1.00		16	1.00		16	1.00	
	PCR	Positive	2	0.13	0.004**	1	0.06	0.001**	0	1.00	0.000**
		Negative	14	0.88		15	0.94		16	1.00	
		Total	16	1.00		16	1.00		16	1.00	
Buffalo	Microscopic	Positive	1	0.06	0.001**	2	0.13	0.004**	1	0.06	0.001**
		Negative	15	0.94		14	0.88		15	0.94	
		Total	16	1.00		16	1.00		16	1.00	
	PCR	Positive	0	1.00	0.000**	0	1.00	0.000**	0	1.00	0.000**
		Negative	16	1.00		16	1.00		16	1.00	
		Total	16	1.00		16	1.00		16	1.00	

NS=Non-significant (p > 0.05),

* *Significant (p < 0.05),

** Highly significant (p < 0.001), PCR=Polymerase chain reaction

**Table-4:** Binomial test (proportion test) for comparison between positive and negative categories with respect to small ruminants and methods for different districts regarding prevalence.

Animal	Methods	Category	Muzaffargarh			Lodhran			Bahawalpur		
		
			Prevalence	Proportion	p-value	Prevalence	Proportion	p-value	Prevalence	Proportion	p-value
Goat	Microscopic	Positive	3	0.19	0.021*	4	0.25	0.077^NS^	2	0.13	0.004**
		Negative	13	0.81		12	0.75		14	0.88	
		Total	16	1.00		16	1.00		16	1.00	
	PCR	Positive	2	0.13	0.004**	1	0.06	0.001**	1	0.06	0.001**
		Negative	14	0.88		15	0.94		15	0.94	
		Total	16	1.00		16	1.00		16	1.00	
Sheep	Microscopic	Positive	5	0.31	0.210^NS^	1	0.06	0.001**	3	0.19	0.021*
		Negative	11	0.69		15	0.94		13	0.81	
		Total	16	1.00		16	1.00		16	1.00	
	PCR	Positive	1	0.06	0.001**	0	1.00	0.000**	0	1.00	0.000**
		Negative	15	0.94		16	1.00		16	1.00	
		Total	16	1.00		16	1.00		16	1.00	

NS=Non-significant (p> 0.05),

* Significant (p < 0.05),

** Highly significant (p < 0.001), PCR=Polymerase chain reaction

## Discussion

This study aimed to estimate the prevalence of trypanosome infections in key ruminant species, including camels, cattle, buffaloes, goats, and sheep, in Southern Punjab, Pakistan. Parasitic diseases pose significant challenges to the global livestock industry, affecting human health, trade, and economies [19]. Trypanosomiasis, as one of the prevalent parasitic disease, has been reported globally, affecting regions such as Africa, Europe, the US, and Asia, resulting in substantial economic losses [20]. Notably, its prevalence extends to countries like Egypt [21], Sudan [22], Somalia [23], Saudi Arabia [24], Iran [25] and Iraq [26, 27], with reported cases in Nigeria [28], Ethiopia [29], Kenya [30], and Jordan [31].

Two hundred and forty blood samples were collected from camels, cattle, buffaloes, goats, and sheep for analysis from three districts in Southern Punjab, Pakistan. Diagnostic tests such as thin blood smear microscopy and PCR were used to detect the presence of *Trypanosoma* spp. in the animals. These tests have a proven track record for detecting *Trypanosoma* spp. A study by Gadahi *et al*. [32] has confirmed the reliability of these tests for detecting parasites like *Trypanosoma* spp., which was essential for estimating the prevalence of trypanosomiasis in the study population. PCR stands out due to its ability to confirm microbial presence while also characterizing it at the subgenus, species, or strain level [32]. The precision of diagnosis in livestock populations is enhanced through PCR’s dual functionality: its ability to both detect and quantify specific genetic material. This dual functionality allows PCR to identify the presence of *Trypanosoma* spp. with high sensitivity and specificity, as well as to provide quantitative data on parasite load, which is crucial for understanding the severity and epidemiology of infections in livestock.

This study employed the TBR 1/2 set of primers to detect trypanosome infections across various animal species, demonstrating its heightened sensitivity. This aligns with a previous study by Pruvot *et al*. [33], which used six sets of primers, including TBR1/2, ESAG6/7, TEPAN1/2, pMUTEC F/R, TRYP1 R/S, and TRYP4 R/S, to confirm different dilutions of the *T. evansi* genome in infected rats and Thai dairy cattle. Among these primers, TBR1/2 demonstrated the highest sensitivity, detecting as little as 0.01 pg of *T. evansi* DNA. In the current study, the prevalence of trypanosome infections varied significantly across livestock species, with camels exhibited the highest rates. These findings highlight the differential susceptibility of species to *T. evans*i infection. The prevalence rates observed in Southern Punjab align with findings from a previous study in Sindh, Pakistan [34], where camels showed a notable prevalence of 13.7%.

The higher prevalence observed in the study area could be attributed to inadequate veterinary practices and variations in environmental conditions, which may contribute to the transmission and persistence of trypanosome infections. Furthermore, this detection method has been successfully used to identify *T. evansi* prevalence in Nili-Ravi buffaloes, aligning with the findings on prevalence rate estimated at 5.5% in Okara district, Pakistan [35]. In another study conducted in Lyari, Karachi, Pakistan [36], cases of trypanosomiasis in donkeys were recorded using electron microscopy, revealed a prevalence rate of 8.4%.

Studies reported from other parts of the world also provide further evidence supporting the efficacy of microscopy in detecting *Trypanosoma* spp. infections across different regions and animal species; In Algeria, a prevalence rate of 14.0% in camels has been reported [37]. Similarly, in Egypt and Sudan, prevalence rates of 4.14% and 1.7%, respectively, were observed [38, 39]. Other studies have reported varying prevalence rates in different livestock populations; For instance, a study in Sudan reported a prevalence of 43% in cattle [40], while in Brazil, a prevalence of 9.1% was observed in cattle [41]. In West Atacora, a region in Benin, thin smear microscopy revealed 67% of cases of *Trypanosoma* infection in cattle and 3.8% in sheep [42]. In Eastern Zambia, infection rates were reported as 13.5%, 0%, and 0.9% in cattle, goats, and pigs, respectively [43]. Similarly, in India, microscopy analysis detected a prevalence of 3.27% of camel samples, while no infection was found in samples from donkeys and dogs [44].

In the realm of PCR diagnostics, the effectiveness of various primers in detecting *Trypanosoma* spp. was investigated, with TBR primer set emerging as the most sensitive in the current study. The use of TBR primers revealed infection rates of 12.50% in camels, 6.25% in cattle, 0.00% in buffaloes, 8.33% in goats, and 2.08% in sheep. Similar results have been reported globally, highlighting the versatility of PCR in *Trypanosoma* spp. diagnosis. For instance, in Sudan, PCR techniques were employed in both high- and low-prevalence areas, yielding prevalence rates of 57.1% and 6.0%, respectively [40]. In Egypt, the NRP1 and NRP2 primers were used, identifying a prevalence of 56.9% in camels [37]. In addition, in Sudan, the TBR1 and TBR2 primers reported a prevalence rate of 90.0% in camels [38].

The study concludes that trypanosomiasis is notably prevalent in southern Punjab, as indicated by microscopic examination with prevalence rates of 23.75% in Muzaffargarh, 21.25% in Lodhran, and 21.25% in Bahawalpur. PCR is recognized as the superior technique for diagnosing *Trypanosoma* spp. due to its high specificity and sensitivity. It effectively minimizes false positives by accurately detecting and identifying the pathogen’s genetic material, especially in regions where multiple species are implicated in trypanosomiasis. Despite the global presence of more than 20 *Trypanosoma* species in the Southeast Asian context, including this study, primarily involves *T. evansi*, *Trypanosoma lewisi*, and *Trypanosoma brucei* [45–47].

Comparatively, findings from Zahoor *et al*. [48], which employed multiple diagnostic methods, including microscope examination with Giemsa staining, formol gel test, and PCR, further supported the observed prevalence of trypanosomiasis. Their study reported a prevalence of 22.5% during microscopic examination, 21% during the formol gel test, and 15.5% during PCR. The lower prevalence rate detected by PCR reflects its higher specificity and ability to reduce false positives rather than a diminished capacity to detect infections. This variance in prevalence rates highlights the strengths and limitations of each method. The study underscores the sensitivity, robustness, and reliability of PCR in diagnosing trypanosomiasis, advocating its incorporation into conventional setups alongside microscopy to reduce false negative and positive results. While microscopy is effective in detecting *Trypanosoma* species, it lacks specificity in identifying the particular species, leading to a higher percentage of positive results compared with PCR. PCR offers a precise tool for discerning specific *Trypanosoma* species, particularly in cases of mixed infections where specificity is crucial.

Based on PCR, the prevalence order of trypanosomiasis in Southern Punjab livestock was as follows: camels > goats > cattle > sheep > buffaloes. Similarly, thin smear microscopy yielded a prevalence order of camels > cattle > goats > sheep > buffaloes.

## Conclusion

This study offers molecular insights into the present occurrence of trypanosomiasis in Southern Punjab’s livestock. Traditional microscopy, when used alongside PCR, brought to light the greater sensitivity and accuracy of PCR for diagnosing different *Trypanosoma* species. According to PCR results, the most common trypanosome species among camels, goats, cattle, sheep, and buffaloes is *T. evansi*. The seasonal fluctuations in *T. evansi* detection demonstrate the infection’s inherent dynamism. This study recommends the implementation of PCR-based screening across Pakistan, combined with vector eradication and farmer education. Reducing the economic impact of trypanosomiasis in the region necessitates the implementation of certain strategies.

## Authors’ contributions

MT and FB: Designed the study. MT, FB, and MSK: Methodology. EIA, MSK, NUK, GAM, MBS, AM, SN, MI, and GAM: Software and validation. MT, EIA, PRDR-E, and FB: Formal analysis. MBS, MSK, MM, AM, NUK, and RT: Investigation. AM, GAM, MSK, MM, NUK, EIA, and FB: Data curation. MT, FB, and MBS: Writing original draft preparation. FB and MBS: Writing review and editing. MM, EIA, NUK, MSK, and AM: Visualization. FB and MBS: Project administration. All authors have read, reviewed, and approved the final version of the manuscript.
